# Nutrients, Food Bioactives, and Functional Foods in Gastrointestinal and Metabolic Disorders

**DOI:** 10.3390/foods14203513

**Published:** 2025-10-15

**Authors:** Lorena Ortega Moreno, Samuel Fernández-Tomé

**Affiliations:** 1 Area of Pharmacology, Nutrition and Food Science, High-Performance Research Group in Digestive System Pathophysiology URJC: NeuGut-URJC, Department of Basic Health Sciences, Rey Juan Carlos University (URJC), 28922 Madrid, Spain; lorena.ortega@urjc.es; 2 Department of Nutrition and Food Science, Faculty of Pharmacy, Complutense University of Madrid (UCM), Plaza Ramón y Cajal s/n, 28040 Madrid, Spain

Gastrointestinal and metabolic disorders represent a major public health burden worldwide. Beyond pharmaceutical treatments, nutrition and functional foods are increasingly recognized as cornerstones for prevention and management strategies. A growing body of research has been focused on dietary bioactive compounds because of their potential ability to modulate glucose and lipid metabolism, inflammation, oxidative stress, gut microbiota composition, and gut barrier integrity [1,2,3].

The Special Topic “Nutrients, Food Bioactives, and Functional Foods in Gastrointestinal and Metabolic Disorders” has gathered 14 outstanding contributions published across different journals, including Foods, Nutrients, Antioxidants, Current Issues in Molecular Biology, and International Journal of Molecular Sciences. These original studies, systematic reviews, and meta-analyses cover a wide spectrum of food bioactives, ranging from resveratrol and isoflavones to plant oils, polysaccharides, peptides, as well as microbial interactions. Collectively, they provided novel mechanistic insights, nutritional mechanisms, and translational implications for nutrition and food science research.

This editorial synthesizes their key findings, discusses integrative themes, and identifies future research directions for functional foods and bioactive compounds in gastrointestinal and metabolic health.

Food bioactives can significantly regulate glycaemic control and insulin sensitivity with strong evidence until now proved for phenolics, peptides and some plant-derived compounds [4,5]. The main mechanisms of action included insulin signaling enhancement, enzyme inhibition, incretin modulation, and both antioxidant and anti-inflammatory effects. Several animal studies from our Special Topic have showed reproducible improvements in glycaemic control and insulin signalling after administration of plant-derived extracts or isolated bioactive phytochemicals, as follows.

Wu et al. (Contribution 1) investigated the flavonoid fraction of *Mao Jian* green tea (MJGT_F) in a streptozotocin/high-fat diet (HFD) rat model of type 2 diabetes mellitus (T2DM). High-dose MJGT_F significantly lowered fasting insulin, improved HOMA-IR, increased the insulin sensitivity index, raised GLP-1 levels, and corrected lipid abnormalities. In vitro, MJGT_F inhibited α-glucosidase activity, while in vivo effects surpassed or matched metformin on several endpoints.

Liu et al. (Contribution 2) explored *Artemisia integrifolia* extract in diabetic animals. Oral administration (90 and 180 mg/kg for 15 days) significantly reduced fasting glucose and improved HOMA-IR, glucose and lipid metabolism, and liver histology, supported by metabolic profiling and molecular docking analysis. Mechanistically, the extract modulated PI3K/AKT signaling, whereas caffeic acid, coumarin, trifolin, and apigetrin were identified as the main bioactive phytochemicals from the extract.

Sulforaphane, a cruciferous-derived isothiocyanate, was studied by He et al. (Contribution 3) in *Helicobacter pylori*-infected mice. Using high-coverage metabolomics and lipidomics, they showed that sulforaphane at 20 mg/kg/day reversed infection-induced metabolic alterations by modulation of glutathione, glycine and serine metabolic pathways, both in serum and liver, highlighting its potential as a dietary strategy against *H. pylori*-associated metabolic dysfunction.

Complementing these preclinical outcomes, Mokgalaboni et al. (Contribution 4) performed a quantitative meta-analysis of randomized clinical trials on curcumin supplementation in T2DM. The pooled data (18 trials and 1382 patients) demonstrated significant improvements in hyperglycaemia and systemic inflammation, compared to placebo, though effect sizes varied by formulation and trial quality, highlighting the need for standardized intervention (time and duration) and prompting further research on optimized absorption and formulations.

Moreover, Renke et al. (Contribution 5) reviewed the potential of resveratrol for treating metabolic and estrogen-dependent disorders. Their review highlighted the dual of this well-known phytochemical in modulating sex hormone pathways and exerting anti-inflammatory actions. Their work reinforced the notion that resveratrol’s pleiotropic activities through activation of estrogen receptors may explain its capacity to address complex metabolic diseases by increased lipolysis and mitigation of oxidative stress and insulin resistance.

A wide range of food bioactives can beneficially affect lipid metabolism and oxidative stress, thus supporting complementary strategies for preventing and managing metabolic diseases [6,7]. The two following animal studies of the Special Topic described notable lipid-lowering and antioxidant effects. Hence, perilla seed oil, a plant-based source rich in omega-3 fatty acids, was tested by Pothinam et al. (Contribution 6) in HFD-hyperlipidemic rats. Supplementation (0.67 g/kg/day for 8 weeks) reduced plasma lipids, and both oxidative stress (malondialdehyde) and systemic inflammation (interleukin-6) biomarkers, suggesting its value in cardiometabolic risk reduction. Boren et al. (Contribution 7) showed that visceral adipose tissue from C3H/HeJ mice fed high-fat diets expressed distinct adipocytokine patterns in terms of adiponectin, leptin, MCP-1, TIMP-1, resistin, and VEGF-A levels, depending on sex, age, and dietary protein type (beef or casein) supplemented with ammonium hydroxide. Their work highlighted the impact of dietary protein processing on adipose tissue inflammation and underscored the complexity of host-diet interactions in metabolic disorders.

Following this line of research, three independent preclinical contributions showed complementary mechanisms of intestinal mucosal protection by reduced inflammation, gut barrier integrity and T-cell signalling modulation. Ulcerative colitis is a chronic immune-mediated disease with no definitive cure. Chen et al. (Contribution 8) reported that early chicken embryonic amniotic fluid alleviated dextran-sulfate-sodium-induced colitis in mice, animal model of human ulcerative colitis, as it decreased TNF-α and IL-1β in colon tissue and increased IL-10. In vitro, gut barrier was also strengthened and authors linked intestinal barrier integrity to adaptive immune modulation and suppression of TCR signaling. This unusual source of bioactive compounds provides new perspectives on immunomodulation by food-derived biologically active compounds [8,9]. Likewise, naringenin, a citrus flavonoid, was shown by Wu et al. (Contribution 9) to promote gastrointestinal motility and the release of digestive hormones in mice. Effects were mediated by modulation of the SCF/c-Kit pathway and microbiota restructuring, suggesting naringenin as a dietary bioactive with potential in functional gastrointestinal disorders. Noteworthy, Chel-Guerrero et al. (Contribution 10) used an isobolographic approach to evaluate the antinociceptive effect of *Salvia hispanica* L. seeds (chia) in combination with lime (*Citrus × latifolia*) in rats by the writhing test. They demonstrated a notable synergistic antinociceptive effect, suggesting novel applications of functional food combinations in inflammation and visceral pain.

Several studies in this Special Topic converge on microbiota modulation as a key mechanism for metabolic and gastrointestinal disorders. Across independent models, this modulation usually leads to increased abundance of beneficial bacteria (e.g., *Bifidobacterium*, *Lactobacillus*, *Akkermansia*…) and reduced levels of pathogenic species, while restoring microbial balance and reducing the dysbiosis usually linked to these physiological alternations [10]. Thus, enhanced production of microbiota-derived metabolites such as short-chain fatty acids, which improve gut barrier integrity, regulate immune responses, and reduce inflammation, are commonly displayed accompanied by amended metabolic phenotypes [11]. Hence, Wu et al. (Contribution 1) reported that MJGT_F extract enriched *Akkermansia muciniphila* and reduced microbial pathogens, correlating flavonoids’s hypoglycemic effects with improved lipid metabolism. Boren et al. (Contribution 7) found that ammoniated protein diets increased *Akkermansia*, *Romboutsia*, and *Oscillospiraceae*, and He et al. (Contribution 3) demonstrated that sulforaphane corrected microbial-host co-metabolic lipid profiles, reinforcing the microbiota–adipose metabolism interplay. Likewise, Wu et al. (Contribution 9) showed that naringenin could also modulate the proportion of microbial communities towards beneficial bacteria such as *Bacteroides acidifaciens.*

Human evidence was also contemplated in the Special Topic by meta-analyses and observational studies. The supplementation with chokeberry (*Aronia melanocarpa*), a polyphenol-rich fruit, for cardiometabolic disorders, was systematically evaluated by Frumuzachi et al. (Contribution 11) in a meta-analysis of randomized controlled trials. By summarizing ten randomized controlled trials including 666 subjects, authors did not find significant pooled effects for body weight, triglycerides, total cholesterol, HDL-C, or blood pressure, even though improved fasting blood glucose level, selected as a cardiometabolic biomarker, was evidenced. Nonetheless heterogeneity in study designs, doses, and formulations, in conjunction with high risk of bias, notably limited the strength of evidence. Furthermore, isoflavones intake in relation to gastric cancer risk was addressed in an epidemiological analysis by Natale et al. (Contribution 12). Their case-control study reported associations between higher isoflavone intake and reduced gastric cancer incidence, with adjusted odds ratios declining across exposure quantiles, although authors reinforced the necessity of replication in prospective cohorts.

Two final methodological contributions were mainly focused on analytical advances, product validation and development. Hence, Benito-Vázquez et al. (Contribution 13) proposed a novel analytical pipeline for characterizing fruit-derived proteolytic nutraceuticals. By application of their pipeline including total soluble protein and proteolytic activity determinations as well as product stability and protein profiling by proteomics, authors identified discrepancies between declared and measured protease activity in commercial products. On the other hand, Bilraheem et al. (Contribution 14) applied analytical optimization of pectin extraction from melon peel, to generate functional hydrolysates with prebiotic activity for three probiotic strains. Beyond their prebiotic potential in vitro, this work provided high yields of structurally defined pectin thereby highlighting the dual benefits of valorizing food by-products into functional ingredients.

Altogether, the collective insights from this Special Topic converge on several unifying topics. First, dietary bioactives frequently demonstrate multifunctionality, meaning single compounds or extracts often influenced glucose and lipid metabolism, oxidative stress, inflammation, and gut barrier integrity simultaneously [12]. The key role of the gut microbiome as central mediator on these studies further strengthens this paradigm [13]. Many interventions improved host physiology in parallel with shifts in microbial composition, diversity and/or function, with enrichment of taxa commonly associated with metabolic health. Such evidence reinforces the concept of a bidirectional diet–microbiome–host axis as frontier in gastrointestinal and metabolic research. Interestingly, clinical and epidemiological human studies highlight promise but also a high complexity. On the other hand, the valorization of agricultural by-products as novel sources of food bioactives, marks a shift toward innovation in sustainable research and functional food development, in alignment with environmental responsibility and circular economy principles in food systems [14].

Despite this encouraging progress, several challenges appeared. Many primary investigations remain preclinical, often conducted in rodent models with small sample sizes and short-term duration. Noteworthy, to enhance intestinal absorption of less bioavailable food bioactives, technological advances such as nanoformulations, encapsulation, and optimized food matrices could be pursued. On the other hand, meta-analyses and observational data indicate protective or corrective effects, although dietary responses are not uniform. Variability in formulations, doses, trial designs, and human responses, point toward the need for harmonization in research protocols. Data from these studies still require cautious interpretation given uncertainties in dose scaling, bioavailability, and interindividual variability in human microbiomes, as well as the vulnerability to possible confounding factors. Another limitation comes from the complexity of reported outcomes from multi-omics; metabolomic, lipidomic, proteomic and microbiome changes are generally described, but causal relationships and systems-level understanding and validation will be necessary to confirm mechanistic pathways and elevate the robustness of the evidence base.

In conclusion, the 14 contributions compiled in this Special Topic highlight the dynamic intersection between nutrition and food research, and digestive and metabolic health. Collectively, they underscore that food bioactives act not only through isolated pathways but also by interconnected networks involving host metabolism, microbial and immune regulation. Preclinical evidence in the field is abundant and mechanistically rich, yet clinical translation remains the key challenge. To bridge this gap, future progress in gastrointestinal and metabolic research will require interdisciplinary and multi-level studies spanning nutrition science, food technology, microbiology, molecular biology, and clinical research.
